# Welcome to the new editor-in-chief

**DOI:** 10.1007/s12471-023-01848-2

**Published:** 2024-01-10

**Authors:** Jan J. Piek

**Affiliations:** Heart Centre, Amsterdam Cardiovascular Sciences, Amsterdam UMC—location AMC, Department of Cardiology, University of Amsterdam, Amsterdam, The Netherlands

Seven years ago, I took over the position of editor-in-chief of the Netherlands Heart Journal from Professor Ernst van der Wall, who had served the journal for 30 years. When I started I expected to act as editor-in-chief for just a few years. However, it takes time to form a new editorial board and further build on Professor van der Wall’s good work.

The Netherlands Heart Journal is among the few journals of national cardiac societies with an impact factor. Our journal is a platform for open discussions on new developments within our profession. We welcome opinion papers to endorse discussion between our members on interpretation of novel diagnostic and therapeutic findings, and also on the application of international guidelines at the national level. The quality of submissions, and in particular the frequently cited and downloaded review articles, has led to an improvement in our impact factor. One of the highlights of the journal was the special issue on cardiac research in the Netherlands, on the occasion of the ESC Congress 2020 in Amsterdam. Unfortunately, this special issue could not be distributed in person, as the ESC meeting was virtual during the COVID pandemic.

During the last year, there was a gradual transition of an old towards a new editorial board and a good timing to step down as editor-in-chief. I am very pleased to welcome Professor Pim van der Harst as the new editor-in-chief. Professor van der Harst is an interventional cardiologist, scientist and head of the Department of Cardiology of the University Medical Center Utrecht. With his background, I am confident that he will lead the new editorial board in a dedicated fashion towards the future. With his team he will contribute to improve the quality of our journal by bringing science and content of our national society together.

I would like to thank the previous deputy editors, Professors Jolanda van der Velden, Ron Peters, Yigal Pinto and Rob de Winter for their dedicated work and Lieda Meester and Lucienne Bongers for their supportive role. Moreover, I wish Professor Pim van der Harst and his team, Dr. Maryam Kavousi, Dr. Peter Damman, Professor Joris de Groot and Dr. Martin Hemels all the best for their esteemed work in the new editorial board.

